# Diagnosis of Tuberculosis by an Enzyme-Linked Immunospot Assay for Interferon-γ

**DOI:** 10.3201/eid1304.051195

**Published:** 2007-04

**Authors:** Jann-Yuan Wang, Chien-Hong Chou, Li-Na Lee, Hsiao-Leng Hsu, I-Shiow Jan, Po-Ren Hsueh, Pan-Chyr Yang, Kwen-Tay Luh

**Keywords:** Tuberculosis, ELISPOT, latent infection, interferon-γ, research

## Abstract

We evaluated an enzyme-linked immunospot assay for interferon-γ (T SPOT-TB) for rapid diagnosis of active tuberculosis (TB) in a disease-endemic area. From January to June 2005, patients whose clinical symptoms and radiographic findings were compatible with TB were recruited, and a blood sample was obtained for T SPOT-TB assay within 7 days of microbiologic studies. Sixty-five patients were studied, including 39 (60%) with active TB. Thirty-five (53.8%) patients had underlying medical conditions. Thirty-seven patients had positive cultures for *Mycobacterium tuberculosis*, and 11 patients had positive cultures for nontuberculous mycobacteria. The sensitivity, specificity, positive predictive value, and negative predictive value of the T SPOT-TB assay were 87.2%, 88.5%, 91.9%, and 82.1%, respectively. The accuracy of this test in diagnosing active TB is >80%, even in an area with a high incidence of nontuberculous mycobacteria disease.

## Abstract 2

*National Taiwan University Hospital, Taipei, Taiwan,

This assay for interferon-γ can rapidly and accurately diagnose active tuberculosis in a disease-endemic area.

Tuberculosis (TB) remains the most important infectious disease in the world. In 2003, the incidence and death rates for TB in Taiwan were 62.38 and 5.80 per 100,000, respectively (*1*). Successful control of TB depends on prompt detection of patients with *Mycobacterium tuberculosis* infection. The conventional methods for laboratory diagnosis of TB, including acid-fast staining and culture, are either insensitive (*2*,*3*) or time-consuming (*4*). Although new diagnostic methods that use nucleic acid amplification and detection may provide quick and specific results for identifying the *M*. *tuberculosis* complex (*5*,*6*), their sensitivities are considerably less than those of culture (*6*).

Recent studies demonstrated that an enzyme-linked immunospot (ELISPOT) assay for interferon-γ (IFN-γ) produced by activated T cells after exposure to antigens of *M*. *tuberculosis*, early secretory antigenic target 6 (ESAT-6), and culture filtrate protein 10 (CFP-10) is a specific method for identifying *M*. *tuberculosis* infection (*7*–*9*). However, its performance in rapid diagnosis of active TB in disease-endemic areas is still unknown. We evaluated the ELISPOT (T SPOT-TB) assay in clinically suspected cases of TB.

## Methods

### Patients

This study was conducted from January to June 2005 in northern Taiwan at a tertiary-care referral center with 2,000 beds; the study was approved by the ethics committee of the hospital. Patients with fever or respiratory symptoms (cough, dyspnea, or hemoptysis) for ≥2 weeks and compatible radiographic findings were considered to have clinically suspected cases of TB. The compatible findings included fibroexudative or fibrotic lesions over upper lung, pulmonary nodules with or without cavitation, multiple patches of alveolar infiltrates, miliary shadowing, and pleural effusion with lymphocytotic and exudative characteristics. Patients were invited to provide informed consent and were interviewed and examined. A blood sample was obtained for ELISPOT within 7 days of microbiologic studies (including acid-fast smears [AFS] and mycobacterial culture). Twelve healthcare workers (HCWs) in the hospital were included as a healthy control group.

### Laboratory Procedures

AFS for respiratory samples and mycobacterial culture were performed as previously described (*5*). If the primary care physician deemed it necessary, samples were screened for infection with HIV type 1 or type 2 viruses by using competitive ELISAs (Wellcome Laboratories, Beckenham, UK), and infection was confirmed by Western blotting (Diagnostics Pasteur, Marnes-la-Coquette, France).

### ELISPOT Assay

Five milliliters of blood was obtained from each patient and processed within 2 hours. ELISPOT was performed by using a commercial kit (T SPOT-TB; Oxford Immunotec Ltd, Oxford, UK) as previously described (*10*). Briefly, peripheral blood mononuclear cells were separated by using Ficoll-Paque centrifugation. Cells were washed, resuspended, and counted. Ninety-six–well polyvinylidene fluoride–backed plates (MAIPS4510; Millipore, Billerica, MA, USA) were coated with 15 μg/mL of monoclonal antibody 1-D1K against IFN-γ (Mabtech, Nacka Strand, Sweden). Cells (250,000/well) were added to duplicate wells containing antigen (ESAT-6 or CFP-10) or mitogen. No antigen was added to the background control wells. After incubation for 18 h, plates were washed, 100 μL (1 μg/mL) of biotinylated monoclonal antibody 7-B6-1-biotin against IFN-γ (Mabtech) was added, and plates were incubated for 2 h. Plates were then washed, streptavidin-alkaline phosphatase toxoid (Mabtech) was added and incubated for 1.5 h; plates were washed again and 100 μL of chromogenic alkaline phosphatase substrate (Bio-Rad Laboratories, Hercules, CA, USA) was added. After 10–15 min, the plates were washed and spots were enumerated with a stereomicroscope independently by 2 observers. Mean values determined by the 2 observers and both duplicate wells were used in all calculations. The number of spots in the background control wells was subtracted from the number in the test wells, and a response was considered positive if the number of spots per test well was ≥10 and at least twice the value found in the background control wells.

### Clinical Evaluation of Patients

All medical records including history, symptoms, signs, radiologic, pathologic, and microbiologic results, and follow-up observations were carefully reviewed to obtain data for generating a clinical diagnosis. On the basis of clinical findings, 2 categories of patients were considered to have active TB: those whose clinical specimens were culture-positive for *M*. *tuberculosis* and those whose biopsy specimens had caseating granulomas that showed marked improvement after treatment.

## Results

Patient characteristics are summarized in Table 1. All patients were previously vaccinated with bacillus Calmette-Guérin (BCG). Laboratory tests for HIV infection were performed for 42 patients and results were positive for 3 patients. Of 23 patients with an unknown HIV status, all had an initial lymphocyte count >1 × 10^9^/L and did not have AIDS-defined illness (*11*). Thirty-nine (60%) had active TB; 37 had culture-confirmed TB and 2 had histopathology-proved TB with marked improvement of their clinical conditions after treatment. Thirty-one patients had pulmonary TB, 3 had TB pleurisy (*M*. *tuberculosis* was isolated from pleural effusion), and 2 had concomitant pulmonary TB and TB pleurisy. The remaining 3 patients had pulmonary and extrapulmonary TB. Of the 26 non-TB patients, all were culture negative for *M*. *tuberculosis* for multiple specimens (mean 8.3, range 3–20).

**Table 1:** Clinical characteristics of 65 patients suspected of having tuberculosis, Taiwan, 2005*

Characteristic	Value
Mean age, y (range)	52.2 (2–84)
Male:female (% male)	37:28 (56.9)
Underlying medical condition	35 (53.8)
Malignancy	17 (26.2)
Diabetes mellitus	12 (18.5)
Other†	11 (16.9)
Symptoms‡	
Cough or dyspnea [no. with hemoptysis]	44 (67.7) [2]
Fever	35 (53.8)
Mean duration of symptoms (range)	23.4 wk (2 wk– 5 y)
Radiographic finding	
Upper lobe fibroexudative lesions [no. with cavitation]	25 (38.5) [2]
Multiple patches of alveolar infiltrates [no. with cavitation]	20 (30.8) [1]
Multiple nodules or mass [no. with cavitation]	10 (15.4) [3]
Upper lobe fibrotic change	5 (7.7)
Pleural effusion [no. with upper lobe fibrotic change]	3 (4.6) [1]
Miliary lesion	2 (3.1)
Acid-fast smear positive	33 (50.8)
Mycobacterial culture	48 (73.8)
*Mycobacterium tuberculosis*	37 (56.9)
*M. avium-intracellulare* complex	2 (3.1)
*M. chelonae*	2 (3.1)
*M. abscessus* and *M. chelonae*	2 (3.1)
*M. abscessus*	1 (1.5)
*M. marinum*	1 (1.5)
*M. kansasii*	1 (1.5)
Unidentified species	2 (3.1)

*Values are no. (%) unless otherwise indicated. †Underlying disease was alcoholism in 4 patients, AIDS in 3 patients, end-stage renal disease in 2 patients, Sjogren syndrome in 1 patient, and hepatitis B-related liver cirrhosis in 1 patient. ‡Two patients were asymptomatic and had abnormal chest radiographs noted incidentally.

Eight (12.3%) fulfilled the diagnostic criteria for nontuberculosis mycobacterial (NTM) disease (*12*). Of these 8 patients, 2 infected with *M*. *avium-intracellulare* complex responded to treatment with clarithromycin, ethambutol, and rifampin; 1 infected with *M*. *kansasii* responded to treatment with isoniazid, rifampin, and ethambutol; and 3 infected with *M*. *abscessus* responded to treatment with clarithromycin. Clinical conditions and radiographic abnormalities improved in 9 patients after treatment with antimicrobial drugs and in 2 patients after treatment with antifungal drugs. Three other patients provided biopsy specimens, which showed malignancy in 2 patients and a benign tumor in 1 patient. Another patient died of *Staphylococcus aureus* pneumonia and bacteremia. Three other patients showed no clinical and radiographic improvement after empiric treatment for 2 weeks. Specimens from 2 these patients were tested by a nucleic acid amplification assay (BD ProbeTec ET DTB system; Becton Dickinson Instrument Systems, Sparks, MD, USA) and showed negative results. Nine of 12 patients with diabetes and the 3 patients infected with HIV had active TB. In the 48 patients with mycobacteria isolated from respiratory specimens, the average interval between the date when microbiologic studies were performed and the date when the result of mycobacterial culture was available was 49.9 days (range 14–77 days). However, the average interval for the ELISPOT assay for these patients was 4.5 days (range 1–8 days) after microbiologic studies were performed.

Table 2 shows the correlation between ELISPOT results and the final diagnosis for the 65 patients. Of the 22 patients with AFS-positive TB, 19 (86.4%) were ELISPOT positive. Three showed false-negative results in the ELISPOT, including a 41-year-old HIV-positive man, a 47-year-old HIV-negative man with diabetes mellitus, and a 78-year-old woman with diabetes mellitus and Sjogren syndrome. Of the 11 non-TB patients with positive AFS, mycobacterial culture showed NTM disease in 8 patients. Three showed false-positive results in the ELISPOT, including a 74-year-old man with diabetes who was culture positive for *M*. *chelonae*, a 50-year-old previously healthy man who was culture positive for *M*. *marinum*, and a 21-year-old previously healthy woman who was culture positive for *M*. *avium-intracellulare* complex. The positive predictive value (PPV) of ELISPOT for AFS-positive patients was 86.4% (Table 2).

**Table 2:** Correlation between results of enzyme-linked immunospot (ELISPOT) assay and diagnosis of 65 patients suspected of having tuberculosis (TB), Taiwan, 2005

Results of acid-fast smears (no. samples)	No. samples					Sensitivity (%)	Specificity (%)	Predictive value (%)	
	TB (n = 39)			No TB (n = 26)					
								Positive	Negative
	ELISPOT (+)	ELISPOT (–)		ELISPOT (+)	ELISPOT (–)				
Positive (33)	19	3		3	8	86.4	72.7	86.4	72.7
Negative (32)	15	2		0	15	88.2	100	100	88.2
Total (65)	34	5		3	23	87.2	88.5	91.9	82.1

Of the 17 patients with AFS-negative TB, 2 (11.8%) showed negative results in the ELISPOT (Table 2). Both were previously healthy and had culture-positive TB pleurisy with pleural effusions with lymphocytotic and exudative characteristics. Chest radiographs for these 2 patients showed pleural effusion without parenchymal lesions. Their sputum cultures were negative for *M*. *tuberculosis*. HIV status was tested in only 1 patient. For the 15 non-TB patients with negative AFS, all showed negative results in the ELISPOT, i.e., the specificity and PPV of the ELISPOT were 100% (Table 2).

Among the 28 ELISPOT-negative patients, 3, including 1 with culture-confirmed TB, were retested 2, 4, and 5 weeks later, respectively. All were again ELISPOT negative. Among the 12 HCWs, all were ELISPOT negative except 1 who previously had culture-confirmed TB and had been treated for 10 months.

## Discussion

Delayed diagnosis and treatment can increase the risk for dissemination of *M*. *tuberculosis* and decrease survival for some subgroups of TB patients (*13*–*15*). Thus, new technologic developments, which facilitate rapid diagnosis, are needed for successful control of this disease. Besides the development of nucleic acid amplification assays for rapid detection of *M*. *tuberculosis* complex, attempts have been made to exploit the T-cell response for rapid diagnosis of *M*. *tuberculosis* infection (*16**,**17*). The major problem with tuberculin skin testing (TST) is cross-reactivity with antigens in other mycobacteria, such as the *M*. *bovis* BCG vaccine strain and environmental mycobacterial species. This cross-reactivity leads to false-positive results and decreased PPV, especially in BCG-vaccinated persons and in areas of high incidence of NTM disease, such as Taiwan. In Taiwan in 2001, 2.74% of preschool children were TST positive, whereas active TB developed in only 2.29/100,000 children 5–9 years of age (*1*). Use of ESAT-6 and CFP-10, two antigens encoded in the region of difference 1, which distinguishes *M*. *tuberculosis* from other mycobacteria, has increased the specificity and PPV of IFN-γ ELISPOT assays (*10*,*18*–*22*). Our study showed that the sensitivity, specificity, PPV, and negative predictive values (NPV) of the ELISPOT assay were >80% in the diagnosis of active TB in clinically suspected patients. Results were also available ≈45 days earlier than those obtained with mycobacterial culture.

The genes coding for ESAT-6 and CFP-10 are absent from most environmental mycobacteria, except for *M*. *kansasii*, *M*. *marinum*, *M*. *szulgai*, *M*. *flavescens*, and *M*. *gastrii* (*23*–*25*). Whether ESAT-6 or CFP-10 is present in *M*. *chelonae* and the *M*. *avium-intracellulare* complex has not yet been determined. Although PPV is associated with pretest probability of active TB in a cohort, our results showed that the ELISPOT can accurately discriminate TB from NTM disease and other respiratory diseases. All 3 patients with false-positive ELISPOT results had NTM disease. The 3 AFS-positive TB patients with false-negative ELISPOT results had other diseases (2 had diabetes mellitus and 1 had AIDS), which could weaken the T-cell response (*26**,**27*). However, neither of the 2 AFS-negative ELISPOT false-negative TB patients had another disease. Because HIV status was not routinely tested, the possibility of asymptomatic HIV infection that potentially influenced the ELISPOT results cannot be excluded.

Consistent with previous reports (sensitivity 80.7%–94.4%) (*20*,*28*–*30*), assays detecting secretion of IFN-γ caused by stimulation with ESAT-6 or CFP-10 for diagnosis of TB have a sensitivity >80%. However, specificity (45.5%–69.2%), PPV (65.4%–85.4%), and NPV (53.6%– 90.0%) were highly variable, which was probably due to different criteria for patient selection and diagnosis of active TB. In a study conducted in Japan (*20*), only patients with culture-confirmed infection were considered to have active TB. Thus, culture-negative TB patients would be classified into a non-TB group, but some showed positive test results, which resulted in decreased specificity and PPV. In the study conducted in Denmark (*28*), several risk factors predisposing persons to recent *M*. *tuberculosis* infection were observed in the 10 patients with false-positive results, including a history of recent exposure, immigration from a highly disease-endemic area, intravenous drug use, and HIV positivity. In the study conducted in Brazil (*30*), controls were medical students, who were at high risk for nosocomial exposure; 50% of them were ELISPOT positive, which resulted in low specificity and PPV.

Many of our patients without active TB were ELISPOT negative. In a study in Taiwan in 2001, 2.74% of preschool children were TST positive, and the annual estimated infection rate was 0.43% (*1*). Therefore, it is unlikely that all of our ELISPOT-negative patients had never been infected with *M*. *tuberculosis*. Furthermore, the results with samples from HCWs decrease the possibility that acute illness caused a false-negative result. Previous studies with sequential testing showed that responses of ESAT-6– or CFP-10–specific T cells decay progressively with treatment for TB (*9*,*22*,*31*–*34*). Our ELISPOT-negative patients may not have been recently infected with *M*. *tuberculosis*; thus, levels of their circulating ESAT-6– or CFP-10–specific effector T cells, rather than memory T cells, decreased and failed to yield a positive ELISPOT result (*35**,**36*). Further long-term follow-up study of ELISPOT-positive TB patients is needed to better understand the dynamic changes in ESAT-6– or CFP-10–specific effector T cells.

Patients with AFS-negative TB should be further investigated because this type of TB is usually diagnosed late and has been reported to be responsible for ≈17% of TB transmission (*37**,**38*). Our study showed that all AFS-negative ELISPOT-positive patients had true cases of TB, i.e., PPV = 100%. Only 2 patients with TB pleurisy and negative sputum culture for *M*. *tuberculosis* showed false-negative ELISPOT results. The cause of this finding is not known because the current hypothesis for the pathogenesis of TB pleurisy is that the caseous material from a subpleural focus ruptures into the pleural space 6–12 weeks after a primary infection. This material then interacts with previously sensitized T cells, which results in a delayed hypersensitivity reaction and accumulation of fluid (*39**-**40*). The 2 patients with false-negative ELISPOT results might have been at a early stage of primary TB, and their sensitized T cells had not yet returned to the systemic circulation before sampling was conducted. Further investigation is needed to assess the performance of the ELISPOT assay in patients suspected of having TB with negative AFS results.

The resurgence of TB has prompted the need for sensitive, accurate, and fast methods for laboratory detection of *M*. *tuberculosis* infection. Although previous studies demonstrated that the ELISPOT assay for INF-γ is a powerful tool for detecting latent *M. tuberculosis* infection, our results showed that in patients who were previously vaccinated with BCG, the diagnostic value of this test in detecting active TB approached 90% in sensitivity, specificity, PPV, and NPV, even in an area with a high incidence of NTM disease.

## References

[1] Center for Disease Control. Statistics of communicable diseases and surveillance report in Taiwan area, 2003. Taipei, Taiwan: Center for Disease Control; 2004.

[2] Boyd JC, Marr JJ. Decreasing reliability of acid-fast smear techniques for detection of tuberculosis. Ann Intern Med. 1975;82:489–92. 1091188 10.7326/0003-4819-82-4-489

[3] Murray PR, Elmore C, Krogstad DJ. The acid-fast stain: a specific and predictive test for mycobacterial disease. Ann Intern Med. 1980;92:512–3. 6153877 10.7326/0003-4819-92-4-512

[4] Pfyffer GE, Brown-Elliott BA, Wallace RJJ. *Mycobacterium*: general characteristics, isolation, and staining procedures. In: Murray PR, Baron EJ, Jorgensen JH, Pfaller MA, Yolken RH, editors. Manual of clinical microbiology, 8th ed. Washington: American Society for Microbiology Press; 2003. p. 532–59.

[5] Wang JY, Lee LN, Chou CS, Huang CY, Wang SK, Lai HC, Performance assessment of a nested-PCR assay (the RAPID BAP-MTB) and the BD ProbeTec ET system for detection of *Mycobacterium tuberculosis* in clinical specimens. J Clin Microbiol. 2004;42:4599–603. 10.1128/JCM.42.10.4599-4603.2004 15472315 PMC522346

[6] Piersimoni C, Scarparo C. Relevance of commercial amplification methods for direct detection of *Mycobacterium tuberculosis* complex in clinical samples. J Clin Microbiol. 2003;41:5355–65. 10.1128/JCM.41.12.5355-5365.2003 14662911 PMC309028

[7] Hill PC, Brookes RH, Fox A, Fielding K, Jeffries DJ, Jackson-Sillah D, Large-scale evaluation of enzyme-linked immunospot assay and skin test for diagnosis of *Mycobacterium tuberculosis* infection against a gradient of exposure in The Gambia. Clin Infect Dis. 2004;38:966–73. 10.1086/382362 15034828

[8] Nicol MP, Pienaar D, Wood K, Eley B, Wilkinson RJ, Henderson H, Enzyme-linked immunospot assay responses to early secretory antigenic target 6, culture filtrate protein 10, and purified protein derivative among children with tuberculosis: implications for diagnosis and monitoring of therapy. Clin Infect Dis. 2005;40:1301–8. 10.1086/429245 15825033

[9] Hill PC, Fox A, Jeffries DJ, Jackson-Sillah D, Lugos MD, Owiafe PK, Quantitative T cell assay reflects infectious load of *Mycobacterium tuberculosis* in an endemic case contact model. Clin Infect Dis. 2005;40:273–8. 10.1086/427030 15655747

[10] Lalvani A, Pathan AA, McShane H, Wilkinson RJ, Latif M, Conlon CP, Rapid detection of *Mycobacterium tuberculosis* infection by enumeration of antigen-specific T cells. Am J Respir Crit Care Med. 2001;163:824–8. 11282752 10.1164/ajrccm.163.4.2009100

[11] Centers for Disease Control and Prevention. 1993 revised classification system for HIV infection and expanded surveillance case definition for AIDS among adolescents and adults. MMWR Recomm Rep. 1992;41(RR-17):1–19. 1361652

[12] Diagnosis and treatment of disease caused by nontuberculous mycobacteria. This official statement of the American Thoracic Society was approved by the Board of Directors, March 1997. Medical Section of the American Lung Association. Am J Respir Crit Care Med. 1997;156:S1–25. 9279284 10.1164/ajrccm.156.2.atsstatement

[13] Alwood K, Keruly J, Moore-Rice K, Stanton DL, Chaulk CP, Chaisson RE. Effectiveness of supervised, intermittent therapy for tuberculosis in HIV-infected patients. AIDS. 1994;8:1103–8. 10.1097/00002030-199408000-00010 7986406

[14] Pablos-Mendez A, Sterling TR, Frieden TR. The relationship between delayed or incomplete treatment and all-cause mortality in patients with tuberculosis. JAMA. 1996;276:1223–8. 10.1001/jama.276.15.1223 8849749

[15] Wang JY, Hsueh PR, Lee LN, Liaw YS, Shau WY, Yang PC, *Mycobacterium tuberculosis* inducing disseminated intravascular coagulation. Thromb Haemost. 2005;93:729–34. 15841321 10.1160/TH04-09-0562

[16] Schluger NW, Rom WN. The host immune response to tuberculosis. Am J Respir Crit Care Med. 1998;157:679–91. 9517576 10.1164/ajrccm.157.3.9708002

[17] Barnes PF. Diagnosing latent tuberculosis infection: the 100-year upgrade. Am J Respir Crit Care Med. 2001;163:807–8. 11282742 10.1164/ajrccm.163.4.ed0201c

[18] Lalvani A, Pathan AA, Durkan H, Wilkinson KA, Whelan A, Deeks JJ, Enhanced contact tracing and spatial tracking of *Mycobacterium tuberculosis* infection by enumeration of antigen-specific T cells. Lancet. 2001;357:2017–21. 10.1016/S0140-6736(00)05115-1 11438135

[19] Shams H, Weis SE, Klucar P, Lalvani A, Moonan PK, Pogoda JM, Enzyme-linked immunospot and tuberculin skin testing to detect latent tuberculosis infection. Am J Respir Crit Care Med. 2005;172:1161–8. 10.1164/rccm.200505-748OC 16081545 PMC2718400

[20] Mori T, Sakatani M, Yamagishi F, Takashima T, Kawabe Y, Nagao K, Specific detection of tuberculosis infection: an interferon-gamma-based assay using new antigens. Am J Respir Crit Care Med. 2004;170:59–64. 10.1164/rccm.200402-179OC 15059788

[21] Brock I, Weldingh K, Lillebaek T, Follmann F, Andersen P. Comparison of tuberculin skin test and new specific blood test in tuberculosis contacts. Am J Respir Crit Care Med. 2004;170:65–9. 10.1164/rccm.200402-232OC 15087297

[22] Lalvani A, Nagvenkar P, Udwadia Z, Pathan AA, Wilkinson KA, Shastri JS, Enumeration of T cells specific for RD1-encoded antigens suggests a high prevalence of latent *Mycobacterium tuberculosis* infection in healthy urban Indians. J Infect Dis. 2001;183:469–77. 10.1086/318081 11133379

[23] Harboe M, Oettinger T, Wiker HG, Rosenkrands I, Andersen P. Evidence for occurrence of the ESAT-6 protein in *Mycobacterium tuberculosis* and virulent *Mycobacterium bovis* and for its absence in *Mycobacterium bovis* BCG. Infect Immun. 1996;64:16–22. 8557334 10.1128/iai.64.1.16-22.1996 PMC173721

[24] Geluk A, van Meijgaarden KE, Franken KL, Subronto YW, Wieles B, Arend SM, Identification and characterization of the ESAT-6 homologue of *Mycobacterium leprae* and T-cell cross-reactivity with *Mycobacterium tuberculosis.* Infect Immun. 2002;70:2544–8. 10.1128/IAI.70.5.2544-2548.2002 11953394 PMC127910

[25] Arend SM, van Meijgaarden KE, de Boer K, de Palou EC, van Soolingen D, Ottenhoff TH, Tuberculin skin testing and in vitro T cell responses to ESAT-6 and culture filtrate protein 10 after infection with *Mycobacterium marinum* or *M. kansasii.* J Infect Dis. 2002;186:1797–807. 10.1086/345760 12447766

[26] Joshi N, Caputo GM, Weitekamp MR, Karchmer AW. Infections in patients with diabetes mellitus. N Engl J Med. 1999;341:1906–12. 10.1056/NEJM199912163412507 10601511

[27] Mukadi YD, Maher D, Harries A. Tuberculosis case fatality rates in high HIV prevalence populations in sub-Saharan Africa. AIDS. 2001;15:143–52. 10.1097/00002030-200101260-00002 11216921

[28] Ravn P, Munk ME, Andersen AB, Lundgren B, Lundgren JD, Nielsen LN, Prospective evaluation of a whole-blood test using *Mycobacterium tuberculosis*-specific antigens ESAT-6 and CFP-10 for diagnosis of active tuberculosis. Clin Diagn Lab Immunol. 2005;12:491–6. 15817755 10.1128/CDLI.12.4.491-496.2005 PMC1074386

[29] Liebeschuetz S, Bamber S, Ewer K, Deeks J, Pathan AA, Lalvani A. Diagnosis of tuberculosis in South African children with a T-cell-based assay: a prospective cohort study. Lancet. 2004;364:2196–203. 10.1016/S0140-6736(04)17592-2 15610806

[30] Abramo C, Meijgaarden KE, Garcia D, Franken KL, Klein MR, Kolk AJ, Monokine induced by interferon gamma and IFN-gamma response to a fusion protein of *Mycobacterium tuberculosis* ESAT-6 and CFP-10 in Brazilian tuberculosis patients. Microbes Infect. 2006;8:45–51. 10.1016/j.micinf.2005.05.019 16269263

[31] Ferrand RA, Bothamley GH, Whelan A, Dockrell HM. Interferon-gamma responses to ESAT-6 in tuberculosis patients early into and after anti-tuberculosis treatment. Int J Tuberc Lung Dis. 2005;9:1034–9. 16158897

[32] Pathan AA, Wilkinson KA, Klenerman P, McShane H, Davidson RN, Pasvol G, Direct ex vivo analysis of antigen-specific IFN-gamma-secreting CD4 T cells in *Mycobacterium tuberculosis*–infected individuals: associations with clinical disease state and effect of treatment. J Immunol. 2001;167:5217–25. 11673535 10.4049/jimmunol.167.9.5217

[33] Sousa AO, Wargnier A, Poinsignon Y, Simonney N, Gerber F, Lavergne F, Kinetics of circulating antibodies, immune complex and specific antibody-secreting cells in tuberculosis patients during 6 months of antimicrobial therapy. Tuber Lung Dis. 2000;80:27–33. 10.1054/tuld.1999.0230 10897381

[34] Carrara S, Vincenti D, Petrosillo N, Amicosante M, Girardi E, Goletti D. Use of a T cell-based assay for monitoring efficacy of antituberculosis therapy. Clin Infect Dis. 2004;38:754–6. 10.1086/381754 14986262

[35] Dheda K, Udwadia ZF, Huggett JF, Johnson MA, Rook GA. Utility of the antigen-specific interferon-gamma assay for the management of tuberculosis. Curr Opin Pulm Med. 2005;11:195–202. 10.1097/01.mcp.0000158726.13159.5e 15818179

[36] Lalvani A. Counting antigen-specific T cells: a new approach for monitoring response to tuberculosis treatment? Clin Infect Dis. 2004;38:757–9. 10.1086/381763 14986263

[37] Behr MA, Warren SA, Salamon H, Hopewell PC, Ponce de Leon A, Daley CL, Transmission of *Mycobacterium tuberculosis* from patients smear-negative for acid-fast bacilli. Lancet. 1999;353:444–9. 10.1016/S0140-6736(98)03406-0 9989714

[38] Hernandez-Garduno E, Cook V, Kunimoto D, Elwood RK, Black WA, FitzGerald JM. Transmission of tuberculosis from smear negative patients: a molecular epidemiology study. Thorax. 2004;59:286–90. 10.1136/thx.2003.011759 15047946 PMC1763818

[39] Stead WW, Eichenholz A, Stauss HK. Operative and pathologic findings in twenty-four patients with syndrome of idiopathic pleurisy with effusion, presumably tuberculous. Am Rev Tuberc. 1955;71:473–502. 14361964 10.1164/artpd.1955.71.4.473

[40] Ferrer J. Pleural tuberculosis. Eur Respir J. 1997;10:942–7. 9150338

